# The Impact of *MGMT* Promoter Methylation and Temozolomide Treatment in Serbian Patients with Primary Glioblastoma

**DOI:** 10.3390/medicina55020034

**Published:** 2019-02-01

**Authors:** Nikola Jovanović, Tatjana Mitrović, Vladimir J. Cvetković, Svetlana Tošić, Jelena Vitorović, Slaviša Stamenković, Vesna Nikolov, Aleksandar Kostić, Nataša Vidović, Miljan Krstić, Tatjana Jevtović-Stoimenov, Dušica Pavlović

**Affiliations:** 1 Department of Biology and Ecology, Faculty of Sciences and Mathematics, University of Niš, 18000 Niš, Serbia; tatjanamitrovic3@gmail.com (T.M.); biovlada@yahoo.com (V.J.C.); tosicsvetlana59@yahoo.com (S.T.); jelena.rajkovic@gmail.com (J.V.); sslavisa@pmf.ni.ac.rs (S.S.); 2 Faculty of Medicine, Clinic of Neurosurgery, Clinical Center, University of Niš, 18000 Niš, Serbia; v.novak@yahoo.com (V.N.); aleko018@yahoo.co.uk (A.K.); 3 Faculty of Medicine, Pathology and Pathological Anatomy Center, University of Niš, 18000 Niš, Serbia; vidovic.patologija@gmail.com (N.V.); krsticmiljan@gmail.com (M.K.); 4 Faculty of Medicine, Institute of Biochemistry, University of Niš, 18000 Niš, Serbia; tjevtovic@yahoo.com (T.J.-S.); pavlovic.dusica@gmail.com (D.P.)

**Keywords:** glioblastoma, *MGMT* methylation, temozolomide, overall survival, prognosis, methylation-specific polymerase chain reaction (MSP)

## Abstract

*Background and objective:* Despite recent advances in treatment, glioblastoma (GBM) remains the most lethal and aggressive brain tumor. A continuous search for a reliable molecular marker establishes the methylation status of the O6-methylguanine-DNA methyltransferase (*MGMT*) gene promoter as a key prognostic factor in primary glioblastoma. The aim of our study was to screen Serbian patients with primary glioblastoma for an *MGMT* promoter hypermethylation and to evaluate its associations with overall survival (OS) and sensitivity to temozolomide (TMZ) treatment. *Materials and methods:* A cohort of 30 Serbian primary glioblastoma patients treated with radiation therapy and chemotherapy were analyzed for *MGMT* promoter methylation and correlated with clinical data. *Results:* *MGMT* methylation status was determined in 25 out of 30 primary glioblastomas by methylation-specific PCR (MSP). *MGMT* promoter hypermethylation was detected in 12 out of 25 patients (48%). The level of *MGMT* promoter methylation did not correlate with patients’ gender (*p* = 0.409), age (*p* = 0.536), and OS (*p* = 0.394). Treatment with TMZ significantly prolonged the median survival of a patient (from 5 to 15 months; *p* < 0.001). *Conclusions:* Due to a small cohort of primary GBM patients, our study is not sufficient for definitive conclusions regarding the prognostic value of *MGMT* methylation for the Serbian population. Our preliminary data suggest a lack of association between *MGMT* promoter methylation and overall survival and a significant correlation of TMZ treatment with overall survival. Further population-based studies are needed to assess the prognostic value of the *MGMT* promoter methylation status for patients with primary glioblastoma.

## 1. Introduction

Glioblastoma (GBM)—World Health Organization (WHO) grade IV diffuse glioma—represents the highly invasive and infiltrative type of primary brain tumor associated with poor prognosis and a 5.6% five-year survival rate [1,2,3]. GBM is the most common type of malignant central nervous system tumor in adults (47.7%–49%) that accounts for the majority of gliomas (56.6%) according to a recent Central Brain Tumor Registry of the United States (CBTRUS) Statistical Report and EUROCARE-5 study [4]. Comprehensive genomic characterization studies revealed an underlying complex network of different molecular aberrations which provoke GBM development through changes in major signaling pathways [5,6]. These studies also contributed toward defining the methylation status of the O^6^-methylguanine-DNA methyltransferase (*MGMT*) gene promoter as one of the most relevant prognostic markers in GBM patients [7,8,9,10,11].

The *MGMT* gene encodes a DNA-repair protein that removes cytotoxic alkyl adducts from O^6^-guanine [12]. This protein inhibits the effect of cancer treatment with alkylating agents such as nitrosoureas, tetrazines, and procarbazine that induce apoptosis in cancer cells [12,13,14]. The alkylating agent Temozolomide (TMZ) was approved in 2005 by the US Food and Drug Administration (FDA) for use in the treatment of GBM [15,16]. TMZ is an imidazotetrazine derivative of decarbazin that induces cell cycle arrest at G2/M. In Serbia, a GBM treatment protocol that includes TMZ as adjuvant therapy was introduced in 2011 [17,18]. Although it was demonstrated that TMZ improves the overall survival (OS) and progression-free survival (PFS) of GBM patients, at least 50% of them do not benefit from TMZ due to treatment resistance caused by over-expression of *MGMT* in GBM cells [19,20]. To date, the bulk of evidence suggests that epigenetic silencing of the *MGMT* gene through hypermethylation of the cytidine phosphate guanosinedinucleotides (CpG) in the promoter region is associated with greater response to the TMZ treatment of GBM patients [15,21,22,23,24]. 

A methylation-specific polymerase chain reaction (MSP) is one of the most commonly used methods for assessing the *MGMT* methylation status in either snap-frozen GBM tissue samples or formalin-fixed, paraffin-embedded (FFPE) tissue [25,26,27,28]. This method is based on sodium bisulfite treatment of isolated DNA samples which results in the conversion of unmethylated cytosines into uracil, leaving methylated cytosines unchanged. Bisulfite conversion of template DNA is followed by PCR reactions using two primer sets for both an unmethylated and methylated *MGMT* promoter variant, which allow for the evaluation of the methylation status at six to nine CpG sites [28,29]. The difference in amplicon lengths after conducting PCR reactions with primer sets for each variant of *MGMT* promoter provides easy-to-interpret results that can be visualized by agarose gel electrophoresis. Since MSP was established, this method has evolved as the “gold standard” that enables a cost-efficient non-quantitative method of *MGMT* methylation analysis suitable for routine clinical diagnostics with low sample numbers [27]. 

The main goal of our study was to determine *MGMT* promoter methylation and its relevance for the prediction and prognosis of clinical outcomes of the Serbian population with glioblastoma. The study was designed to investigate the effect of novel therapeutic treatment (TMZ) on overall survival. Also, the potential use of MSP as a semi-quantitative method for assessing *MGMT* methylation status in snap-frozen GBM samples was investigated. 

## 2. Materials and Methods 

### 2.1. Patients and Tumor Specimens

GBM patients operated on the Neurosurgery Clinic (The Clinical Centre of Niš, Serbia) between 2013 and 2017 were included in this study. All patients underwent total resection of the tumor and had a Karnofsky score ≥80%. Tumor specimens were snap frozen and stored at −80 °C. All samples were confirmed with glioblastoma WHO grade IV by an expert neuropathologist (N.V. and M.K.). The study protocol and informed consent form were approved by the Ethics Committee of the Faculty of Medicine, Niš, Serbia (01-2113-10). Written informed consent was obtained from all study participants. All patients received combined radiotherapy and chemotherapy. Patients were irradiated with 3D conformal radiotherapy at a dosage of 60 Gy in 30 fractions (2 Gy per day, 5 days a week) (radiotherapy (RT)). Patients were classified into three groups based on chemotherapy administered in 6 cycles:

Group 1 (*n* = 10 patients): temozolomide (TMZ)—the first cycle at a dose of 150 mg/m^2^ for 5 days; the next 5 cycles at a dose of 200 mg/m^2^. Cycles were repeated every 3 weeks.
Group 2 (*n* = 10 patients): procarbazine, lomustine (1-[2-chloroethyl]-3-cyclohexyl-1-chloroethylnitrosourea (CCNU)) and vincristine (PCV regimen): CCNU 110 mg/m^2^ p.o. day 1; procarbazine 60 mg/m^2^ per os (p.o.) days 8–21; vincristine 1.4 mg/m^2^ (maximum 2 mg), i.e., days 8 and 21. Cycles were repeated every 6–8 weeks.
Group 3 (*n* = 10 patients): carmustine (BCNU) 200 mg/m^2^, i.e., day 1. Cycles were repeated every 8 weeks.

Although 30 patients were enrolled in this study, DNA was successfully obtained for only 25 samples, see Table 1. For 5 patients, we did not have sufficient tissue specimen for DNA analysis (2 from Group 2 and 3 from Group 3 of treatment). During DNA isolation and PCR analysis, we conducted blind-experiments without knowledge of patients’ diagnosis and treatment (tumor specimens were coded). 

### 2.2. DNA Isolation and Bisulfite Conversion

Genomic DNA was extracted from frozen tumor tissues by QIAamp^®^ DNA Mini Kit (Qiagen, Hilden, Germany) [27]. Quantity and quality of isolated DNA was determined by a BioSpec–nano UV–Vis Spectrophotometer (Shimadzu, Kyoto, Japan). A total of 2 µg of genomic DNA was modified by sodium bisulfite using EpiTect^®^ Bisulfite Kit (Qiagen, Hilden, Germany). 

### 2.3. Methylation-Specific Polymerase Chain Reaction (MSP)

**Figure 1:**
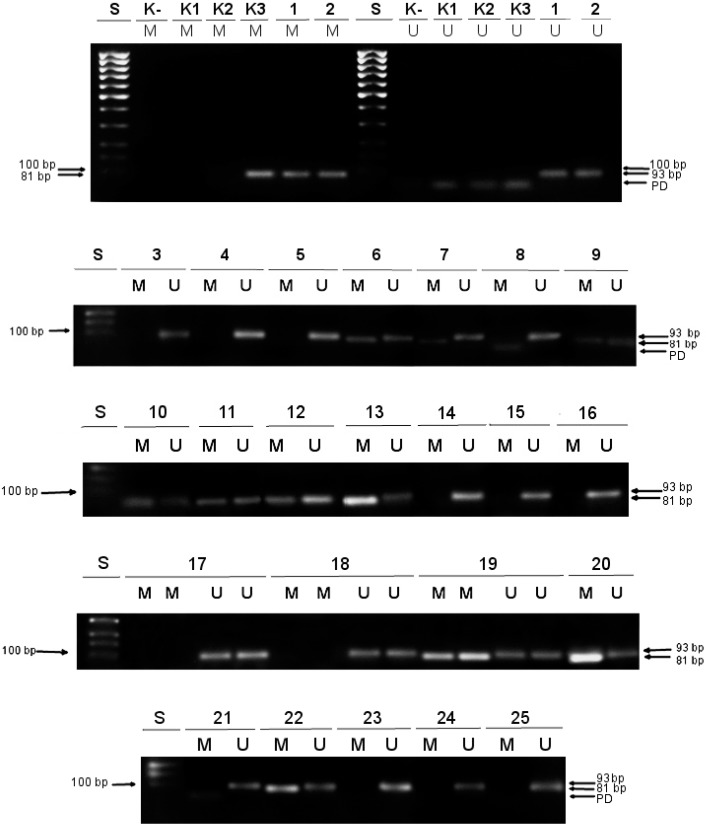
Determination of the methylation status of *MGMT* promoter in primary glioblastoma by A methylation-specific polymerase chain reaction (MSP). S: DNA standard 100 bp ladder; K-: negative control; K1: unmethylated human control DNA; K2: unmethylated and bisulfite-converted human control DNA; K3: methylated and bisulfite-converted human control DNA; M: Polymerase chain reaction (PCR) reaction with primers specific for methylated *MGMT* promoter; U: PCR reaction with primers specific for unmethylated *MGMT* promoter; PD: primer dimers; 1–25: bisulfite-converted DNA isolated from patients with primary glioblastoma; patients denoted as 1, 2, 5, 8, 9, 15, 16, 17, 19, and 25 were treated with RT+TMZ (defined as Group 1 in Section 2.1); patients marked as 4, 7, 10, 12, 18, 22, 23, and 24 were treated with RT+PCV (defined as Group 2 in Section 2.1); patients designated as 3, 6, 11, 13, 14, 20, and 21 were treated with RT+BCNU (defined as Group 3 in Section 2.1.)

The MSP was conducted in a total volume of 20 µL containing 1 × PCR buffer with 1.5 mM MgCl_2_ (Qiagen, Hilden, Germany), 10 pM of appropriate forward and reverse primer, 0.2 μM dNTP mix, 1U HotStar Taq polymerase (Qiagen, Hilden, Germany), and 100 ng of bisulfite-converted template DNA. Primers used for amplification of MGMT promoter and control *ALU–C4* sequences are shown in Table 2. The amplification reaction was carried out in a Mastercycler Gradient (Eppendorf) using the following program: 95 °C for 15 min, then 35 cycles of 95 °C for 50 s, 59 °C for 50 s and 72 °C for 50 s, and final extension at 72 °C for 10 min. Control PCR reactions were performed using EpiTect PCR Control DNA set (Qiagen, Hilden, Germany) consisting of:

- unmethylated and unconverted human DNA (genomic DNA purified from a human colorectal cancer cell line HCT116 DKO with double knockouts of both DNA methyltransferases (DNMT1 (-/-) and DNMT3b (-/-)) (K1 in Figure 1);
- unmethylated and bisulfite-converted human DNA (genomic DNA originated from the same HCT116 DKO cell line as K1 DNA, but modified by sodium bisulfite upon isolation; as a result of bisulfite conversion non-methylated cytosines were turned to uracils) (K2 in Figure 1);
- methylated and bisulfite-converted human DNA (genomic DNA derived from HCT116 DKO cell line which was in vitro methylated at all cytosine positions comprising CpG dinucleotides by *M.SssI* methyltransferase and then treated with sodium bisulfite; the final outcome of the bisulfite treatment was that 5-methylcytosines were left unaffected) (K3 in Figure 1).

The function of these control DNAs in MSP were as follows; while K1 served as negative control in MSP with M or U primers (independently) and for assessment of the efficiency of bisulfite-mediated conversion of DNA, K2 was used as a positive control in MSP with U primers specified for non-methylated cytosines, and K3 was used as a positive control in MSP with M primers specified for 5-methylated cytosines in CpG dinucleotides of MGMT promoter.

**Figure 2:**
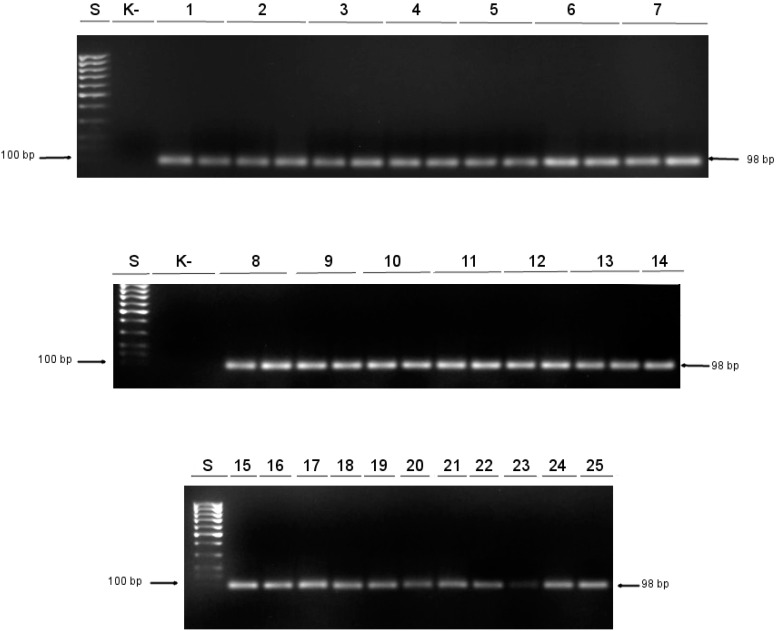
Amplification of a part of the *ALU* element (*ALU C4* sequence) was used for the normalization of MSP. S: DNA standard 100 bp ladder; K-: negative control; 1–25: bisulfite-converted DNA isolated from patients with primary glioblastoma.

Also, a non-template PCR reaction was included as a negative (water) control of PCR (K− in Figure 1 and Figure 2).

*ALU*–based control reaction was used as a control reaction to measure input DNA levels and normalized the signal for each methylation reaction (*ALU–C4* in Table 2 and Figure 2). 

All PCR reactions were performed in duplicate.

Amplified PCR products were detected by ultraviolet (UV) light on a 2% agarose gel stained with ethidium bromide. A visible M primer band of *MGMT* indicated a positive *MGMT* methylation status, while the absence of an M primer PCR product was considered as a negative methylation status of *MGMT.* A visible U primer band of *MGMT* indicated the presence of unmethylated *MGMT* promoter [26]. Primer dimerization was noticed in PCR reactions with U primer (PD in Figure 1).

Gel images were subject to ImageJ software analysis (National Institute of Health, Bethesda, MD, USA) [30].

### 2.4. Quantification of Methylation Data

The level of methylated DNA (percentage of methylated reference (PMR)) was calculated by three different approaches. The first approach compared the intensity of methylated (M) and unmethylated (U) MSP bands on agarose gel using the following formula [27]:

(1) PMR= M/U

The other two approaches for MSP quantification included two control PCR products: *ALU–C4* (*ALU*) as a DNA input normalizer and commercial methylated bisulfite-converted human DNA (Qiagen) as a fully methylated control [32,33]. Equations used for these two approaches were:

(2) $$PMR=\frac{\;M/U\;/\;ALU\;\mathrm{for}\;\mathrm{sample}}{\;M/U\;/\;ALU\;\mathrm{for}\;\mathrm{methylated}\;\mathrm{control}}$$

and

(3) $$PMR=\frac{\;M\;/\;ALU\;\mathrm{for}\;\mathrm{sample}\;}{\;M/\;ALU\;\mathrm{for}\;\mathrm{methylated}\;\mathrm{control}}$$

where in all three approaches for the quantification of MSP: PMR > 1 indicates a strong *MGMT* promoter methylation (hypermethylated), PMR = 0 (no M primer MSP product detectable) indicates an unmethylated *MGMT* promoter and PMR < 1 indicates weak *MGMT* promoter methylation.

### 2.5. Statistical Analysis

Statistical analyses were performed using the SPSS 16.0 software package (IBM Corp., Armonk, NY, USA) with *p* < 0.05 considered significant. Continuous data were presented as mean ± standard variation while categorical data were shown as frequencies (%). Fisher’s exact test was used to test the association between categorical variables and a Student’s *t*-test was used to compare continuous variables.

The patient analysis included gender, age, Karnofsky performance status, methylation status, treatment with TMZ, and survival. Overall survival (OS) was measured from the date of surgery to the date of death or last follow-up. OS curves were estimated by the Kaplan–Meier method and their comparison was performed with the use of a univariate log-rank test. In order to compare the three variants of PMR for assessment of the *MGMT* methylation status, the interclass correlation coefficient (ICC) was determined.

## 3. Results

### 3.1. Methylation Status of the MGMT Promoter and Clinical Parameters

DNA obtained from 25 patients with primary glioblastoma was subjected to MSP with specific primers for methylated (M) and unmethylated (U) template detection. Methylation data were successfully determined for all GBM samples, see Figure 1. Control PCR reactions with *ALU* primers for every specimen were done simultaneously, see Figure 2.

Characteristics of patients within the study group (6 females, 19 males; age 59.6 ± 13.07; 29 to 80 years old) and their methylation status are shown in Table 3. A positive methylation status was detected in 12 patients (48%). Statistical analysis did not find a significant correlation between *MGMT* promoter methylation and gender (χ^2^ = 0.680; *p* = 0.409) or the age of patients with primary GBM (*t* = 0.629; *p* = 0.536).

### 3.2. Different Approaches in MSP Data Quantification

Methylation levels were estimated by three different approaches. The first assessed *MGMT* promoter methylation by a simple M/U ratio for each tumor specimen (PMR (I)). The other two approaches allowed better discrimination between *MGMT* methylation levels in different samples by the inclusion of the PCR signal of a commercial fully methylated control and *ALU* DNA input control (PMR (II) and (III), respectively). Results are shown in Table 4.

*MGMT* promoter methylation status evaluated as PMR (I) and (III) showed identical distribution among patients (five patients with M/U ratio <1 and seven patients with M/U ratio >1), while PMR (II) had different pattern (six patients with M/U ratio <1 and six patients with M/U ratio >1).

Levels of coincidence between various PMR approaches are shown in Table 5. PMR (II) and PMR (III) variants of MSP data demonstrated the highest level of coincidence (ICC = 0.844), while the lowest level of coincidence was between PMR (I) and PMR (III).

### 3.3. MGMT Status, TMZ Therapy, and Survival

**Figure 3:**
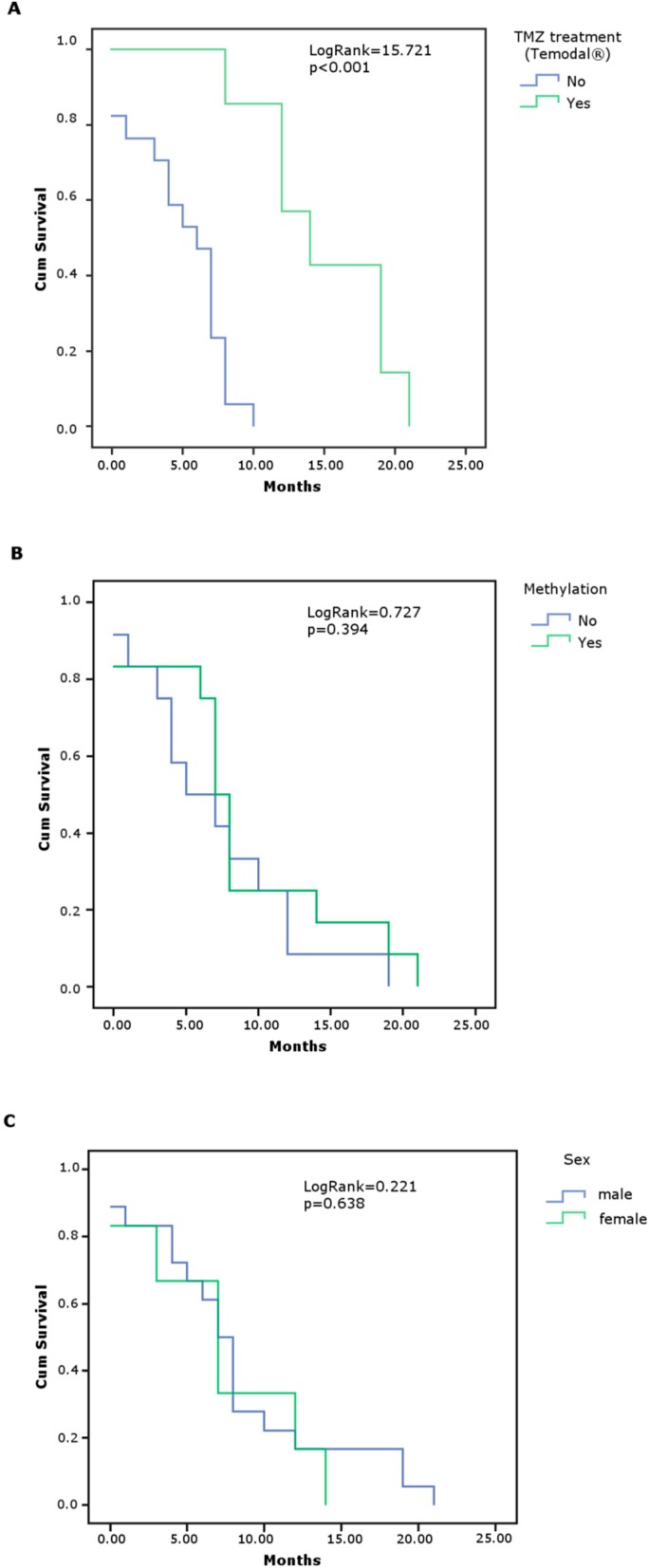
Kaplan–Meier estimates of overall survival (months) related to temozolomide treatment (**A**), methylation status of *MGMT* promoter (**B**), and gender (**C**). Overall Survival: time from date of surgery to death or the end of the follow-up. Cum Survival: cumulative survival as the proportion of surviving in time. *p*: probability value calculated using log-rank test. *p* value <0.05 were considered statistically significant.

Univariate analyses showed that TMZ-treated patients had a statistically significant improvement in overall survival (median survival 15 months) in comparison with patients without TMZ treatment (median survival five months) (*p* < 0.001), see Table 6. It was found that this improvement was not associated with the methylation status of the *MGMT* promoter or gender. Kaplan–Meier OS curves are shown in Figure 3.

## 4. Discussion 

There is ongoing debate concerning the most suitable technique for the determination of the *MGMT* promoter methylation and the prognostic importance of the obtained methylation status for patients with GBM [28,34]. MGMT testing in our study is performed by MSP as one of the oldest and the most widely used techniques [25,26,27,28]. Notably, MSP is cost-effective, gel-based, and the most appropriate method for resource-limited settings and routine diagnostics with low sample numbers. However, this technique is especially prone to producing false-positive results when performed on low quality/quantity DNA, partially bisulfite-converted DNA, or tumor specimens with irregular mosaic methylation patterns [28]. Generally, only vital (non-necrotic) tumor specimens should be used for MSP analysis to avoid false-negative results [28].

In order to improve MSP semi-quantitative potentials, we performed additional normalization of the methylation signal by *ALU* control and universal positive methylation control [32,33]. Therefore, we compensate PMR for variations in copy number due to differences in sample handling, DNA isolation and tumor heterogeneity. Optimally standardized and easy-to-interpret MSP data were used in our study for evaluation of the clinical importance of the methylation status of the *MGMT* promoter.

Further, numerous GBM clinical trials with TMZ have established a positive methylation status of the *MGMT* promoter as the strongest predictor for OS and progression-free survival (PFS) benefit [13,19,23,27,35]. However, our study showed no significant impact of the *MGMT* promoter methylation on the survival outcome and TMZ treatment benefit. Although, we should emphasize that these are only preliminary data based on low sample quantity. Nevertheless, the same observation was made in the above-mentioned study of 110 GBM patients from Serbia; although, the methylation status was assessed in only 62 patients (56.4%) of the cohort [17].

Controversial observations about the predictive and prognostic value of *MGMT* promoter methylation were noted in several studies [14,36] and in meta-analysis [13]. Jesien-Lewandowicz et al. (2009) detected a positive methylation status in 23 out of 32 (72%) primary GBM patients from Poland treated with surgery and radiotherapy [14]. In univariate analysis, the presence of *MGMT* promoter methylation was not associated with the patient’s gender and longer survival. Kalkan and colleagues (2015) assessed *MGMT* promoter methylation status on 40 primary glioblastoma from Turkish patients [36]. They found positive methylation in 13 samples (32.5%) and no statistical significance between *MGMT* methylation and gender and overall survival.

Intratumoral and temporal heterogeneity may underlie the described discrepancies in our and other studies with negative prognostic values of the *MGMT* status [37]. Alternatively, negative conclusions in *MGMT* studies with Polish, Turkish, and Serbian GBM patients may reflect population molecular differences in gliomagenesis. Although, we should mention that these are small size studies which require confirmation in larger-scale, prospective controlled trials. Previously, Wiencke et al. (2005) showed a substantial ethnic specificity of molecular features (*MGMT*, *TP53* and *EGFR*) in 556 glioma samples in the San Francisco Bay Area [38].

Our study has several limitations. First, it was conducted on small cohorts of patients from a single Clinical Centre in Serbia and the obtained results should be interpreted with care. Therefore, we could not definitively rule out the prognostic value of the *MGMT* promoter methylation status in the Serbian GBM population. Second, only the independent prognostic value of *MGMT* methylation was considered. Although the study was carefully performed, the complexity of gliomagenesis and the latest WHO classification of glioma 2016, suggested that the combination of *MGMT*, *IDH1*, and/or *TP53* analysis is more relevant for the prediction of survival of patients with GBM [2]. 

The significance of the combination of predictive biomarkers rather than their individual status for survival prediction in patients with GBM was demonstrated widely [39,40,41,42]. Meta-analysis of Zou and colleagues suggested that *IDH* mutations were tightly associated with *MGMT* promoter hypermethylation (*p* < 0.001) and *TP53* gene mutation (*p* < 0.001) [39]. They indicated that the *IDH* mutation rate was linked to the glioma’s genomic profile. Higher rates of G to A transitions in *IDH1* codon 132 and *TP53* codons 248 and 273 were explained by higher levels of methylation of the *MGMT* promoter CpG islands [39,40]. These mutational events were considered as early events in gliomagenesis which might affect a common stem glial precursor cell population. They were linked with a low proliferation tumor phenotype and a favorable prognosis in glioma patients. Similarly, Shamsara et al. (2009) detected hypermethylation of the *MGMT* promoter in 24 out of 50 patients (48%) and mutation of *TP53* gene in 26 out of 50 patients (52%) with primary glioblastoma in Iran [41]. A significant association between *MGMT* methylation status and *TP53* mutation status was found (*p <* 0.05). *TP53* mutations were observed in 17 out of 26 patients (65.4%) with *MGMT*-hypermethylated glioblastoma. Likewise, in the previously mentioned study of Jesien-Lewandowicz and associates, the frequency of TP53 G:C to A:T mutations were higher in patients with *MGMT* promoter methylation (6 out of 23 patients (26%), *p* = 0.376) [14]. Further, Wang et al. (2014) investigated the predictive value of the combination of *MGMT* methylation status and *TP53* and *IDH1* mutation status in 78 patients with GBM from China [42]. For patients with *IDH1* mutation, *MGMT* hypermethylation was correlated with better overall survival (*p* = 0.013), while for the patients without *IDH1* mutation, the presence of *TP53* mutation was associated with improved survival (*p* = 0.029).

A remarkable improvement in the overall survival of GBM patients is recorded from 2005 since the approval of TMZ for concomitant treatment with radiotherapy (RT) and adjuvant treatment for newly diagnosed GBM [15,22,24]. Meta-analysis of survival outcomes of newly diagnosed GBM patients revealed that the RT + TMZ-treated group of patients had a significantly higher median survival (13.41–19 months) in comparison with RT-alone group (7.7–17.1 months) [22]. 

In Serbia, TMZ was introduced in 2011. Recent studies suggested that TMZ treatment had a favorable impact on the overall survival of GBM patients in Serbia [17,18]. In comparison with RT + BCNU/CCNU treatment, the overall survival of TMZ treated patients was significantly higher (the first study 19 months vs. 13 months; the second study 14.79 months vs. 9. 91 months) [17,18]. Our study confirmed previous findings regarding the favorable impact of TMZ treatment on OS of GBM patients in Serbia (15 months vs. 5 months).

## 5. Conclusions

In contrast to the generally accepted attitude of the prognostic significance of *MGMT* promoter methylation in GBM patients, our study failed to show its prognostic value. Our preliminary data suggest the absence of a prognostic implication of *MGMT* promoter methylation and confirm TMZ treatment benefit on the survival outcome of patients with primary GBM in Serbia. The present small cohort study cannot be used for definitive conclusions and demands independent confirmation in larger population-based studies. Furthermore, elucidation of the true importance of *MGMT* methylation status in primary GBM requires its association with other markers (*IDH1*, *TP53*, etc.)

## Figures and Tables

**Table 1:** List of patients and treatments involved in DNA analysis.

Type of Therapy	Patient Mark
RT + TMZ (Group 1)	1, 2, 5, 8, 9, 15, 16, 17, 19 and 25
RT + PCV (Group 2)	4, 7, 10, 12, 18, 22, 23 and 24
RT + BCNU (Group 3)	3, 6, 11, 13, 14, 20 and 21

RT: radiotherapy; TMZ: temozolomide; PCV: procarbazine, lomustine, and vincristine; BCNU: carmustine.

**Table 2:** Primer sequences and amplification.

Gene	Primer Sequence (5’–3’)	Amplicon Size (bp)	References
*MGMT* unmethylated (U)	F: TTTGTGTTTTGATGTTTGTAGGTTTTTGT	93	[26]
	R: AACTCCACACTCTTCCAAAAACAAAACA		
*MGMT* methylated (M)	F: TTTCGACGTTCGTAGGTTTTCGC	81	[26]
	R: GCACTCTTCCGAAAACGAAACG		
*ALU–C4*	F: GGTTAGGTATAGTGGTTTATATTTGTAATTTTAGTA	98	[31]
	R: ATTAACTAAACTAATCTTAAACTCCTAACCTCA		

MGMT: O^6^-methylguanine-DNA methyltransferase; F: forward primer; R: reverse primer.

**Table 3:** Clinical characteristics and methylation status of primary glioblastoma (GBM) patients.

		Unmethylated (*n* = 13)	Methylated (*n* = 12)
Sex, *n* (%)	male	9 (69.2)	10 (83.3)
	female	4 (30.8)	2 (16.7)
Age, mean ± SD, years		58.00 ± 12.85	61.33 ± 13.65
Age, *n* (%)	<50 yr	4 (30.8)	2 (16.7)
	>50 yr	9 (69.2)	10 (83.3)
Preoperative KPS, (%)		81.64 ± 12.01	81.64 ± 12.01
Postoperative KPS, (%)		80.00 ± 12.06	80.00 ± 12.06

KPS: Karnofsky performance status.

**Table 4:** Semi-quantitative evaluation of the level of methylated *MGMT* promoter using different approaches ((I) to (III)); see Material and Methods).

PMR	(I)	(II)	(III)
<1, *n* (%)	5 (20.0)	6 (24.0)	5 (20.0)
>1, *n* (%)	7 (28.0)	6 (24.0)	7 (28.0)
0, *n* (%)	13 (52.0)	13 (52.0)	13 (52.0)

**Table 5:** Correlation of MSP data obtained by various percentage of methylated reference (PMR) approaches.

	ICC	95% CI	*p*
(I) vs. (II)	0.763	0.532–0.888	<0.001
(I) vs. (III)	0.493	0.139–0.739	0.005
(II) vs. (III)	0.844	0.678–0.928	<0.001

ICC: interclass correlation coefficient; CI: Confidence interval.

**Table 6:** Associations between overall survival, TMZ treatment, gender and MGMT methylation assessed by univariate analyses (log-rank test).

	$\mathrm{Overall}\;\mathrm{Survival}\;(\mathrm{Months})\;\bar{x}$	SE	95% CI		Log-Rank	*p*
			Lower Limit	Upper Limit		
No TMZ	5.000	0.781	3.469	6.531	15.721	<0.001
TMZ	15.000	1.799	11.473	18.527		
Male	8.167	1.462	5.300	11.033	0.221	0.638
Female	7.167	2.151	2.950	11.383		
Unmethylated *MGMT*	7.083	1.574	3.999	10.168	0.727	0.394
Methylated *MGMT*	8.750	1.855	5.114	12.386		

$\bar{x}$: mean value; SE: standard error; CI: confidence interval; *p*: probability value calculated using log-rank test; *p* values < 0.05 were considered statistically significant.

## References

[1] Louis D.N., Ohgaki H., Wiestler O.D., Cavenee W.K., Burger P.C., Jouvet A., Scheithauer B.W., Kleihues P. (2007). The 2007 WHO Classification of Tumours of the Central Nervous System. Acta Neuropathol..

[2] Louis D.N., Perry A., Reifenberger G., von Deimling A., Figarella-Branger D., Cavenee W.K., Ohgaki H., Wiestler O.D., Kleihues P., Ellison D.W. (2016). The 2016 World Health Organization Classification of Tumors of the Central Nervous System: A summary. Acta Neuropathol..

[3] Ostrom Q.T., Gittleman H., Truitt G., Boscia A., Kruchko C., Barnholtz-Sloan J.S. (2018). CBTRUS Statistical Report: Primary Brain and Other Central Nervous System Tumors Diagnosed in the United States in 2011–2015. Neuro Oncol..

[4] Visser O., Ardanaz E., Botta L., Sant M., Tavilla A., Minicozzi P., Hackl M., Zielonke N., Oberaigner W., Van Eycken E. (2015). Survival of adults with primary malignant brain tumours in Europe; Results of the EUROCARE-5 study. Eur. J. Cancer.

[5] Brennan C.W., Verhaak R.G.W., McKenna A., Campos B., Noushmehr H., Salama S.R., Zheng S., Chakravarty D., Sanborn J.Z., Berman S.H. (2013). The Somatic Genomic Landscape of Glioblastoma. Cell.

[6] McLendon R., Friedman A., Bigner D., Van Meir E.G., Brat D.J., Mastrogianakis G.M., Olson J.J., Mikkelsen T., Lehman N., The Cancer Genome Atlas Research Network (2008). Comprehensive genomic characterization defines human glioblastoma genes and core pathways. Nature.

[7] Anvari K., Seilanian Toussi M., Ayatollahi H., Bahadorkhan G., Ghavam Nasiri M., Fazl Ersi M. (2018). Prognostic Significance of MGMT Promoter Methylation in Patients with Glioblastoma Undergoing Surgical Intervention: A Retrospective Study in Northeastern Iran. Middle East J. Cancer.

[8] Arora I., Gurav M., Rumde R., Dhanavade S., Kadam V., Kurani H., Shetty O., Goda J., Shetty P., Moiyadi A. (2018). MGMT gene promoter methylation and its correlation with clinicopathological parameters in glioblastomas. Neurol. India.

[9] Miranda A., Blanco-Prieto M., Sousa J., Pais A., Vitorino C. (2017). Breaching barriers in glioblastoma. Part I: Molecular pathways and novel treatment approaches. Int. J. Pharm..

[10] Li H., Li J., Cheng G., Zhang J., Li X. (2016). IDH mutation and MGMT promoter methylation are associated with the pseudoprogression and improved prognosis of glioblastoma multiforme patients who have undergone concurrent and adjuvant temozolomide-based chemoradiotherapy. Clin. Neurol. Neurosurg..

[11] Pala A., Schmitz A.L., Knoll A., Schneider M., Hlavac M., König R., Wirtz C.R., Coburger J. (2018). Is MGMT promoter methylation to be considered in the decision making for recurrent surgery in glioblastoma patients?. Clin. Neurol. Neurosurg..

[12] Esteller M., Hamilton S.R., Burger P.C., Baylin S.B., Herman J.G. (1999). Inactivation of the DNA Repair Gene O6-Methylguanine-DNA Methyltransferase by Promoter Hypermethylation is a Common Event in Primary Human Neoplasia. Cancer Res..

[13] Binabaj M.M., Bahrami A., ShahidSales S., Joodi M., Joudi Mashhad M., Hassanian S.M., Anvari K., Avan A. (2018). The prognostic value of MGMT promoter methylation in glioblastoma: A meta-analysis of clinical trials. J. Cell. Physiol..

[14] Jesien-Lewandowicz E., Jesionek-Kupnicka D., Zawlik I., Szybka M., Kulczycka-Wojdala D., Rieske P., Sieruta M., Jaskolski D., Och W., Skowronski W. (2009). High incidence of MGMT promoter methylation in primary glioblastomas without correlation with TP53 gene mutations. Cancer Genet. Cytogenet..

[15] Lee S.Y. (2016). Temozolomide resistance in glioblastoma multiforme. Genes Dis..

[16] Woo P., Ho J., Lam S., Ma E., Chan D., Wong W.-K., Mak C., Lee M., Wong S.-T., Chan K.-Y. (2018). A Comparative Analysis of the Usefulness of Survival Prediction Models for Patients with Glioblastoma in the Temozolomide Era: The Importance of Methylguanine Methyltransferase Promoter Methylation, Extent of Resection, and Subventricular Zone Location. World Neurosurg..

[17] Ilic R., Somma T., Savic D., Frio F., Milicevic M., Solari D., Nikitovic M., Lavrnic S., Raicevic S., Milosevic S. (2017). A Survival Analysis with Identification of Prognostic Factors in a Series of 110 Patients with Newly Diagnosed Glioblastoma Before and After Introduction of the Stupp Regimen: A Single-Center Observational Study. World Neurosurg..

[18] Nikolov V., Stojanović M., Kostić A., Radisavljević M., Simonović N., Jelenković B., Berilazić L. (2018). Factor affecting the survival of patients with glioblastoma multiforme. J. BUON.

[19] Meng W., Jiang Y., Ma J. (2017). Is the prognostic significance of O6-methylguanine- DNA methyltransferase promoter methylation equally important in glioblastomas of patients from different continents? A systematic review with meta-analysis. Cancer Manag. Res..

[20] Tini P., Pastina P., Nardone V., Sebaste L., Toscano M., Miracco C., Cerase A., Pirtoli L. (2016). The combined EGFR protein expression analysis refines the prognostic value of the MGMT promoter methylation status in glioblastoma. Clin. Neurol. Neurosurg..

[21] De Carlo E., Gerratana L., De Maglio G., Buoro V., Cortiula F., Gurrieri L., Isola M., Fasola G., Puglisi F., Pizzolitto S. (2018). Defining a prognostic score based on O6-methylguanine-DNA methyltransferase cut-off methylation level determined by pyrosequencing in patients with glioblastoma multiforme. J. Neurooncol..

[22] Feng E., Sui C., Wang T., Sun G. (2017). Temozolomide with or without Radiotherapy in Patients with Newly Diagnosed Glioblastoma Multiforme: A Meta-Analysis. Eur. Neurol..

[23] Stupp R., Mason W.P., van den Bent M.J., Weller M., Fisher B., Taphoorn M.J.B., Belanger K., Brandes A.A., Marosi C., Bogdahn U. (2005). Radiotherapy plus Concomitant and Adjuvant Temozolomide for Glioblastoma. N. Engl. J. Med..

[24] Zhu P., Du X.L., Lu G., Zhu J.-J. (2017). Survival benefit of glioblastoma patients after FDA approval of temozolomide concomitant with radiation and bevacizumab: A population-based study. Oncotarget.

[25] Herman J.G., Graff J.R., Myöhänen S., Nelkin B.D., Baylin S.B. (1996). Methylation-specific PCR: A novel PCR assay for methylation status of CpG islands. Proc. Natl. Acad. Sci. USA.

[26] Esteller M., Garcia-Foncillas J., Andion E., Goodman S.N., Hidalgo O.F., Vanaclocha V., Baylin S.B., Herman J.G. (2000). Inactivation of the DNA-Repair Gene MGMT and the Clinical Response of Gliomas to Alkylating Agents. N. Engl. J. Med..

[27] Christians A., Hartmann C., Benner A., Meyer J., von Deimling A., Weller M., Wick W., Weiler M. (2012). Prognostic value of three different methods of MGMT promoter methylation analysis in a prospective trial on newly diagnosed glioblastoma. PLoS ONE.

[28] Cankovic M., Nikiforova M.N., Snuderl M., Adesina A.M., Lindeman N., Wen P.Y., Lee E.Q. (2013). The Role of MGMT Testing in Clinical Practice. JMD.

[29] Parrella P., la Torre A., Copetti M., Valori V.M., Barbano R., Notarangelo A., Bisceglia M., Gallo A.P., Balsamo T., Poeta M.L. (2009). High specificity of quantitative methylation-specific PCR analysis for MGMT promoter hypermethylation detection in gliomas. J. Biomed. Biotechnol..

[30] Image Processing and Analysis in Java Home page. https://imagej.nih.gov/ij/.

[31] Aithal M.G.S., Rajeswari N. (2015). Validation of housekeeping genes for gene expression analysis in glioblastoma using quantitative real-time polymerase chain reaction. Brain Tumor Res. Treat..

[32] Rezvani N., Alibakhshi R., Vaisi-Raygani A., Bashiri H., Saidijam M. (2017). Detection of SPG20 gene promoter-methylated DNA, as a novel epigenetic biomarker, in plasma for colorectal cancer diagnosis using the MethyLight method. Oncol. Lett..

[33] Håvik A.B., Brandal P., Honne H., Dahlback H.-S.S., Scheie D., Hektoen M., Meling T.R., Helseth E., Heim S., Lothe R.A. (2012). MGMT promoter methylation in gliomas-assessment by pyrosequencing and quantitative methylation-specific PCR. J. Transl. Med..

[34] Dullea A., Marignol L. (2016). MGMT testing allows for personalised therapy in the temozolomide era. Tumor Biol..

[35] Stupp R., Hegi M.E., Mason W.P., van den Bent M.J., Taphoorn M.J.B., Janzer R.C., Ludwin S.K., Allgeier A., Fisher B., Belanger K. (2009). Effects of radiotherapy with concomitant and adjuvant temozolomide versus radiotherapy alone on survival in glioblastoma in a randomised phase III study: 5-year analysis of the EORTC-NCIC trial. Lancet Oncol..

[36] Kalkan R., Atli E.İ., Özdemir M., Çiftçi E., Aydin H.E., Artan S., Arslantaş A. (2015). IDH1 mutations is prognostic marker for primary glioblastoma multiforme but MGMT hypermethylation is not prognostic for primary glioblastoma multiforme. Gene.

[37] Parker N.R., Khong P., Parkinson J.F., Howell V.M., Wheeler H.R. (2015). Molecular heterogeneity in glioblastoma: Potential clinical implications. Front. Oncol..

[38] Wiencke J.K, Aldape K., McMillan A., Wiemels J., Moghadassi M., Miike R., Kelsey K.T., Patoka J., Long J., Wrensch M. (2005). Molecular Features of Adult Glioma Associated with Patient Race/Ethnicity, Age, and a Polymorphism in *O*^6^-Methylguanine-DNA-Methyltransferase. Cancer Epidemiol. Biomark. Prev..

[39] Zou P., Xu H., Chen P., Yan Q., Zhao L., Zhao P., Gu A. (2013). IDH1/IDH2 mutations define the prognosis and molecular profiles of patients with gliomas: A meta-analysis. PLoS ONE.

[40] Ohgaki H., Kleihues P. (2007). Genetic pathways to primary and secondary glioblastoma. Am. J. Pathol..

[41] Shamsara J., Sharif S., Afsharnezhad S., Lotfi M., Raziee H.R., Ghaffarzadegan K., Moradi A., Rahighi S., Behravan J. (2009). Association Between MGMT Promoter Hypermethylation and p53 Mutation in Glioblastoma. Cancer Invest..

[42] Wang K., Wang Y., Ma J., Wang J., Li S., Jiang T., Dai J. (2014). Prognostic value of MGMT promoter methylation and TP53 mutation in glioblastomas depends on IDH1 mutation. Asian Pac. J. Cancer Prev..

